# Association Studies of Calcium-Sensing Receptor (CaSR) Polymorphisms with Serum Concentrations of Glucose and Phosphate, and Vascular Calcification in Renal Transplant Recipients

**DOI:** 10.1371/journal.pone.0119459

**Published:** 2015-03-18

**Authors:** Valerie N. Babinsky, Fadil M. Hannan, Sonia C. Youhanna, Céline Maréchal, Michel Jadoul, Olivier Devuyst, Rajesh V. Thakker

**Affiliations:** 1 Academic Endocrine Unit, Radcliffe Department of Medicine, Oxford Centre for Diabetes, Endocrinology and Metabolism (OCDEM), University of Oxford, Oxford, United Kingdom; 2 Institute of Physiology, Zurich Center for Integrative Human Physiology (ZIHP), University of Zurich, Zurich, Switzerland; 3 Division of Nephrology, Cliniques universitaires Saint-Luc, Université Catholique de Louvain, Brussels, Belgium; University of Bari Aldo Moro, ITALY

## Abstract

**Background:**

Cardiovascular disease is the major cause of death in renal transplant recipients (RTRs) and linked to arterial calcification. The calcium-sensing receptor (CaSR), a G-protein coupled receptor, plays a pivotal role in extracellular calcium homeostasis and is expressed in the intimal and medial layers of the arterial wall. We investigated whether common *CASR* gene variants are predictors for aortic and coronary artery calcification or influence risk factors such as serum calcium, phosphate and glucose concentrations in RTRs.

**Methods:**

Two hundred and eighty four RTRs were investigated for associations between three *CASR* promoter region single nucleotide polymorphisms (SNPs) (rs115759455, rs7652589, rs1501899), three non-synonymous *CASR* coding region SNPs (A986S, R990G, Q1011E), and aortic and coronary artery calcium mass scores, cardiovascular outcomes and calcification risk factors that included serum phosphate, calcium, total cholesterol and glucose concentrations.

**Results:**

Multivariate analysis revealed that RTRs homozygous for the minor allele (SS) of the A986S SNP, when compared to those homozygous for the major allele (AA), had raised serum glucose concentrations (8.7±5.4 vs. 5.7±2.1 mmol/L, *P*<0.05). In addition, RTRs who were heterozygous (CT) at the rs115759455 SNP, when compared to those homozygous for the major allele (CC), had higher serum phosphate concentrations (1.1±0.3 vs. 1.0±0.2 mmol/L, *P*<0.05). *CASR* SNPs were not significant determinants for aortic or coronary artery calcification, and were not associated with cardiovascular outcomes or mortality in this RTR cohort.

**Conclusions:**

Common *CASR* SNPs may be independent predictors of serum glucose and phosphate concentrations, but are not determinants of vascular calcification or cardiovascular outcomes.

## Introduction

Cardiovascular disease is the major cause of premature death in renal transplant recipients (RTRs) [1]. Cardiovascular events and mortality in RTRs are strongly linked to the presence of substantial vascular calcification, which affects > 30% of transplanted patients [2]. Vascular calcification is an active disease process characterised by mineral deposition within the medial and intimal layers of the arterial wall [3, 4]. Medial calcification is a consequence of dysregulated systemic mineral homeostasis and associated with the trans-differentiation of vascular smooth muscle cells (VSMCs) in the arterial media to osteochondrocytic cells that release matrix vesicles, which act as a nidus for mineralisation in the presence of elevated circulating calcium and/or phosphate concentrations. [4]. Medial calcification reduces the compliance of large arteries such as the thoracic aorta, thereby leading to hypertension and left ventricular dysfunction [5]. Intimal calcification develops within atherosclerotic plaques, and is the major form of mineral deposition within the coronary arteries [6]. Thus, the presence of intimal calcification is an indicator of advanced atherosclerosis and associated with myocardial infarction [7]. Major risk factors for atherosclerotic plaque development and intimal calcification include elevations in serum total cholesterol and glucose concentrations, together with increased systolic blood pressure [8].

**Fig 1:**
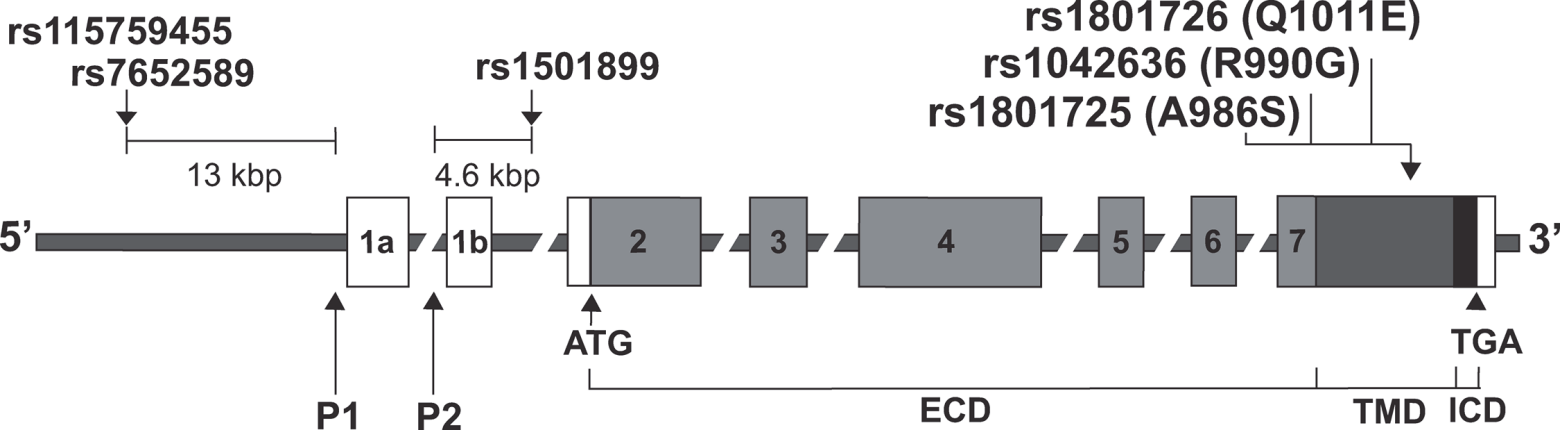
Schematic representation of the genomic organisation of the *CASR* showing location of the six SNPs selected for analysis. The *CASR* gene consists of 8 exons (1a, 1b, 2–7). The start (ATG) and stop (TGA) codons are in exons 2 and 7, respectively. Exons 1a, 1b, the 5’ portion of exon 2, and the 3’ portion of exon 7 are untranslated. The 3’ portion of exon 2, exons 3, 4, 5, 6 and the 5’ portion of exon 7, encode the extracellular domain (light grey), and the mid portion of exon 7 encodes the transmembrane (dark grey) and intracellular (black) domain. Three of the six SNPs are located in the *CASR* promoter region. rs115759455 and rs7652589 are located 13kbp upstream from the TATA box of promoter 1 (P1) and rs1501899 is located 4.6kbp downstream of promoter 2 (P2). The remaining three SNPs (rs1801725, rs1042636 and rs1801726) encoding single amino acid substitutions (A986S, R990G and Q1011E, respectively) are clustered in exon 7. The 5’ and 3’ untranslated regions are shown as white boxes. ECD, extracellular domain; TMD, transmembrane domain; ICD, intracellular domain.

Arterial calcification has been reported to have a substantial genetic component [9, 10], and previous studies have demonstrated associations with common polymorphisms in genes encoding inhibitors of blood vessel mineralisation such as fetuin A and matrix Gla protein [11, 12]. However, the contribution to vessel calcification from polymorphisms of the calcium-sensing receptor (CaSR), which is a G-protein coupled receptor (GPCR) that plays a pivotal role in systemic mineral homeostasis through its effects on parathyroid hormone (PTH) secretion and renal tubular calcium reabsorption [13], has not been investigated. Moreover, the CaSR is expressed and functionally active in the intimal and medial layers of large elastic arteries such as the aorta, and muscular arteries such as the coronary, tibial and internal mammary arteries [14–17], and abnormal functioning of the arterially expressed CaSR has been implicated in the trans-differentiation of VSMCs to mineralising cells, and development of vessel wall calcification [17]. We therefore hypothesised that *CASR* variants may be determinants for arterial medial and intimal calcification, and calcification risk factors that include serum calcium, glucose and phosphate concentrations, in high risk patient groups such as RTRs, and selected six single nucleotide polymorphisms (SNPs) (3 non-synonymous coding region SNPs and 3 promoter region SNPs) (Fig. 1), five of which have been previously associated with indices of mineral metabolism and/or cardiovascular disease [18–22]. We investigated a well characterised cohort of RTRs [2, 23] for associations between these *CASR* SNPs and cardiovascular outcomes, mortality, coronary artery calcification (CAC) and aortic calcification (AoC), and vascular calcification risk factors, which included: systolic blood pressure, serum calcium, phosphate, total cholesterol and glucose concentrations.

## Materials and Methods

### Patients

Patients were ascertained from the Brussels Renal Transplant Cohort [2, 23], which was collected between February 3^rd^ 2004 and January 27^th^ 2005, and comprised >280 RTRs that had an isolated kidney graft functioning for >1 year. The study protocol was approved by The Ethics Committee of the Université catholique de Louvain (UCL) Medical School, Brussels and written informed consent was obtained from all patients [2].

### Clinical parameters

At baseline, clinical parameters including a history of cardiovascular events (defined as myocardial infarction, cerebrovascular event or transient ischaemic attack, and lower limb necrosis or revascularisation) were recorded by a review of the medical charts. Blood samples were obtained at inclusion for biochemical analysis. Serum creatinine, total calcium, phosphate, glucose and total cholesterol concentrations were measured using a Synchron CX analyzer (Beckman Coulter). Serum intact PTH concentrations were measured by a two-site immunoradiometric method (Nichols Institute), and serum 25-hydroxyvitamin D and 1,25-dihydroxyvitamin D concentrations were measured using a LIAISON analyzer (Diasorin Inc). At inclusion, calcification of the aorta and the main coronary arteries were assessed by multi-slice spiral CT scanning of the chest on a 16-slice scanner (Brillance 16; Philips Healthcare, http://www.healthcare.philips.com), as described [2]. The calcium mass of the thoracic aorta and the 4 branches of the main coronary arteries were scored individually, as previously described [2]. Agatston scores and amount of hydroxyapatite (mg) of the coronary arteries and thoracic aorta were measured using a manufacturer algorithm (Heart Beat CS; Philips Healthcare) and by a single operator. Intra-operator variability was 3% and 8%, respectively, for CAC and AoC [2]. Substantial amounts of coronary artery and aortic calcification were defined as >100 mg and >600 mg, respectively, as previously reported [2]. The RTRs were followed up for a mean duration of 4.4 ± 0.3 years and underwent repeat measurement of aortic and coronary artery Agatston scores by spiral CT scanning, and assessment of cardiovascular event incidence and mortality, as described [23].

### Genotyping *CASR* gene polymorphisms

Six *CASR* polymorphisms were selected for analysis. Three SNPs (rs115759455, rs7652589 and rs1501899) were located in the promoter region of the *CASR* and three were non-synonymous SNPs located in the coding region of exon 7 (A986S (rs1801725), R990G (rs1042636) and Q1011E (rs1801726)) (Fig. 1). These six SNPs were genotyped using leukocyte DNA obtained from 284 RTRs, as described [11] and using the following PCR primers (promoter 1 forward: TGAACCTCTACAGCCCTTCG, promoter 1 reverse: GGCAATGTAAAGCGGAAAAA; promoter 2 forward: GTGGTCAGTGAGGGAGAGGA, promoter 2 reverse: GGCATGGAGTGAGGGTACAT; exon 7 forward: CAGAAGGTCATCTTTGGCAGCGGCA, exon 7 reverse: TCTTCCTCAGAGGAAAGGAGTCTGG). All SNPs were analysed by Sanger sequencing of a PCR-product amplified using the BigDye Terminator v3.1 Cycle Sequencing Kit (Life Technologies, Grand Island, NY) and an automated detection system (ABI 3730 Automated capillary sequencer; Applied Biosystems) [24]. Departure from the Hardy-Weinberg equilibrium was determined by Chi-squared analysis (*X* ^2^). Observed SNP allele frequencies in the study cohort were compared to SNP frequencies of the National Heart, Lung and Blood Institute Exome Sequencing Project ((NHLBI-ESP) http://evs.gs.washington.edu/EVS/) and the 1000 Genomes Project (http://www.1000genomes.org/) [25, 26]. Haplotype frequencies were estimated by the maximum likelihood method using Haplotyper software [27]. Linkage disequilibrium was calculated using Haploview v4.2.

### Statistical analysis

Analyses were performed using IBM SPSS version 20 software. Association analyses were conducted between *CASR* SNPs and baseline clinical and biochemical parameters. Radiological parameters measured at baseline and after a mean follow-up period of 4.4 ± 0.3 years were included in the analysis. Parameters showing a right skewed distribution were log transformed prior to parametric analyses. The power of the study was determined using the web-based program QUANTO v1.2 [28]. A univariate analysis of associations was performed using Pearson’s cross product correlation and Chi-squared test for continuous and categorical variables, respectively [11]. All variables that showed associations at the *P* ≤0.2 statistical level, following Bonferroni correction, were entered into a stepwise multivariate linear regression model and an assessment of confounding variables was performed. Kaplan-Meier curves were used to analyse the impact of *CASR* SNPs on all-cause mortality and cardiovascular event free survival and compared by the Mantel (log-rank) test [29]. Results are presented as mean ± SD, or number of patients (N (%)), as appropriate. The results of the multivariate analysis are presented as regression coefficient (B) values ± 95% confidence interval (CI). A value of *P* <0.05 was considered significant for all analyses.

## Results

### Patient characteristics

At baseline, the study cohort comprised 284 adult RTRs (168 males and 116 females) of European origin, with a mean ± SD age of 52.8 ± 12.6 years (Table 1). The patients had a moderate reduction of kidney function (CKD stage II-IIIa), explained by the post-renal transplant status, and the mean concentrations of serum biochemical markers of mineral metabolism, glucose and total cholesterol were within normal limits (Table 1). Hyperparathyroidism (defined as serum PTH concentrations >6.5 pmol/L), hyperphosphataemia (defined as serum phosphate concentrations >1.50 mmol/L) and diabetes affected 25%, 9% and 15% of the study cohort, respectively (Table 1). Baseline assessment of arterial calcification by spiral CT scanning in 266 RTRs revealed substantial amounts of CAC (>100 mg hydroxyapatite) and AoC (>600 mg hydroxyapatite) in >20% and >35% of individuals, respectively (Table 1). Follow-up CAC and AoC spiral CT assessments, after a mean period of 4.4 ± 0.3 years, in 187 individuals revealed >50% of patients to have an increase in CAC or AoC, with 30% of patients having experienced ≥1 cardiovascular events, with an overall mortality rate for the cohort of 12%.

**Table 1:** Baseline clinical, biochemical and radiological characteristics of the Brussels Renal Transplant Cohort.

Parameter	Value
**Clinical (N = 284)**	
**Age (years)**	52.8 ± 12.6
**Gender (males)**	168 (59%)
**Body mass index (kg/m** ^**2**^)	26.4 ± 4.8
**Years after kidney graft**	7.8 ± 6.3
**Parathyroidectomised subjects**	44 (16%)
**Systolic blood pressure (mmHg)**	136 ± 20
**History of smoking**	152 (54%)
**Patients with hyperparathyroidism**	70 (25%)
**Patients with hyperphosphataemia**	26 (9%)
**Patients with diabetes mellitus**	43 (15%)
**Serum (N = 284)** ^**a**^	
**Creatinine (μmol/L)**	122 ± 58
**Estimated glomerular filtration rate (ml/min/1.73 m** ^**2**^)	56 ± 24
**Glucose (mmol/L)**	5.7 ± 2.2
**Total cholesterol (mmol/L)**	5.3 ± 1.1
**Total calcium (mmol/L)**	2.4 ± 0.1
**Phosphate (mmol/L)**	1.0 ± 0.2
**Intact parathyroid hormone (pmol/L)**	5.7 ± 4.6
**25-hydroxyvitamin D (nmol/L)**	42.8 ± 24.4
**1,25-dihydroxyvitamin D (pmol/L)**	86.7 ± 43.3
**Radiological (N = 266)**	
**Aortic calcification score (AgS)**	3309 ± 7101
**Coronary artery calcification score (AgS)**	939 ± 1600
**Number of patients with aortic calcification >600mg**	63 (24%)
**Number of patients with coronary artery calcification >100mg**	99 (37%)

Results are presented as mean ± SD or the number of patients with the % of the total of number of patients shown in parentheses. AgS, Agatston score.

^a^Normal serum ranges: creatinine, 53–124 μmol/L; glucose, 3.8–6.1 mmol/L; total cholesterol, <5.0 mmol/L; total calcium, 2.10–2.50 mmol/L; phosphate, 0.77–1.50 mmol/L; intact parathyroid hormone, 1.0–6.5 pmol/L; 25-hydroxyvitamin D, 75–250 nmol/L; 1,25-dihydroxyvitamin D, 47–117 pmol/L.

### *CASR* SNP frequencies in renal transplant recipients

Six *CASR* SNPs were selected for an assessment of genotype and allele frequencies (Fig. 1 and Table 2). Three of these SNPs are coding region variants (A986S, R990G and Q1011E), and the other three SNPs are located in the promoter region (rs7652589, rs1501899 and rs115759455). The promoter region SNP, rs115759455, has not been previously reported and was detected in this patient cohort during the study. The allelic frequencies of these six *CASR* SNPs (Table 2) were not significantly different to those observed in large Caucasian population cohorts such as the NHLBI-ESP and 1000 Genomes Project cohorts [25, 26]. Furthermore, the allelic frequencies of these six *CASR* SNPs did not deviate from the Hardy-Weinberg equilibrium (Table 2). Linkage disequilibrium was observed between the rs1501899 and rs7652589 promoter region SNPs (*r* ^*2*^ = 0.79), but not between the other *CASR* variants (*r* ^*2*^ <0.015).

**Table 2:** *CASR* polymorphism genotype and allele frequencies in the Brussels Renal Transplant Cohort.

SNP ID	Location	Nucleotide substitution	Amino acid substitution	Genotype frequencies	Allele frequencies	Hardy-Weinberg equilibrium
rs115759455	5’ UTR (c.-83867)	C > T	-	0.91/0.08/0.01	0.95/0.05	X^2^ = 3.13 (*P* = 0.08)
rs7652589	5’ UTR (c.-83707)	G > A	-	0.41/0.44/0.15	0.63/0.37	X^2^ = 1.12 (*P* = 0.28)
rs1501899	5’ UTR (c.-65467)	G > A	-	0.42/0.45/0.13	0.64/0.36	X^2^ = 0.44 (*P* = 0.51)
rs1801725	Exon 7 (c.2956)	G > T	A986S	0.76/0.22/0.02	0.87/0.13	X^2^ = 0.56 (*P* = 0.45)
rs1042636	Exon 7 (c.2968)	A > G	R990G	0.88/0.11/0.01	0.94/0.06	X^2^ = 0.87 (*P* = 0.35)
rs1801726	Exon 7 (c.3031)	C > G	Q1011E	0.93/0.07/0.0	0.97/0.03	X^2^ = 0.38 (*P* = 0.54)

Genotype frequencies are provided for homozygous major alleles, heterozygous alleles, and homozygous minor alleles, respectively in N = 284 subjects. Allelic frequencies are provided for major and minor alleles, respectively. UTR, untranslated region.

### Association of *CASR* SNPs with serum glucose, indices of mineral metabolism, arterial calcification scores and clinical outcomes

Investigations for associations between individual *CASR* variants and arterial calcification scores, systolic blood pressure, serum glucose, serum total cholesterol and serum markers of mineral metabolism (Table 3) revealed an association between *CASR* SNPs and serum metabolites, but not directly with cardiovascular disease. Thus, univariate analysis revealed a significant association between the A986S and serum glucose concentrations (Table 3). The mean glucose concentration of patients that were homozygous for the major allele A986 allele (AA; N = 216) was 5.7 ± 2.1 mmol/L compared with 8.7 ± 5.4 mmol/L for patients that were homozygous for the S986 minor allele (SS; N = 6) (*P* <0.05). All patients were taking prednisolone as part of their immunosuppressant regimen. However, differences in the cumulative dosage of this glucocorticoid medication did not significantly affect serum glucose concentrations in the study cohort (S1 Table).

**Table 3:** Univariate analysis of associations between *CASR* SNP genotypes and risk factors for aortic and coronary artery calcification.

	rs115759455			rs7652589			rs1501899			A986S			R990G			Q1011E		
	**Genotype**	**N**	**Value**	**Genotype**	**N**	**Value**	**Genotype**	**N**	**Value**	**Genotype**	**N**	**Value**	**Genotype**	**N**	**Value**	**Genotype**	**N**	**Value**
**Calcium**	**CC**	259	2.38 ±0.14	**GG**	116	2.38± 0.13	**GG**	120	2.38 ± 0.13	**AA**	217	2.37 ± 0.14	**RR**	251	2.38 ± 0.14	**QQ**	264	2.38 ± 0.14
**(mmol/L)**	**CT**	23	2.39 ± 0.16	**GA**	124	2.37 ± 0.15	**GA**	126	2.36 ± 0.15	**AS**	61	2.39 ± 0.13	**RG**	31	2.35 ± 0.13	**QE**	20	2.4 2± 0.11
	**TT**	2	-	**AA**	43	2.42 ± 0.11	**AA**	38	2.42 ± 0.11	**SS**	6	2.42 ± 0.12	**GG**	2	-	**EE**	0	-
**Phosphate**	**CC**	258	1.00 ±0.23	**GG**	116	1.0 ± 0.2	**GG**	120	1.0 ± 0.2	**AA**	216	1.0 ± 0.3	**RR**	250	1.01±0.25	**QQ**	263	1.0 ± 0.3
**(mmol/L)**	**CT**	**23**	**1.14 ± 0.31**	**GA**	123	1.1 ± 0.3	**GA**	125	1.1 ±0.3	**AS**	61	1.0 ± 0.2	**RG**	31	1.02 ± 0.19	**QE**	20	1.0 ± 0.2
	**TT**	2	-	**AA**	43	0.9 ± 0.2	**AA**	38	1.0 0.2	**SS**	6	0.94 ± 0.1	**GG**	2	-	**EE**	0	-
**PTH**	**CC**	259	5.7 ± 4.6	**GG**	116	5.5 ± 4.1	**GG**	120	5.5 ± 4.0	**AA**	217	5.7 ± 4.9	**RR**	251	5.7 ± 4.6	**QQ**	264	5.7 ± 4.7
**(pmol/L)**	**CT**	23	6.0 ± 4.5	**GA**	124	6.3 ± 5.4	**GA**	126	6.2 ± 5.4	**AS**	61	5.7 ± 3.6	**RG**	31	5.8 ± 4.7	**QE**	20	4.9 ± 2.3
	**TT**	2	-	**AA**	43	4.6 ± 2.9	**AA**	38	4.8 ± 3.1	**SS**	6	4.7 ±4.3	**GG**	2	-	**EE**	0	-
**Creatinine**	**CC**	259	121.3 ± 56.3	**GG**	116	124 ± 63	**GG**	120	123 ± 62	**AA**	217	125.3 ± 62.1	**RR**	251	122.0 ± 59.2	**QQ**	264	122.3 ± 59.7
**(μmol/L)**	**CT**	23	134.3 ± 79.1	**GA**	124	126 ± 60	**GA**	126	126 ± 59	**AS**	61	111.1 ± 43.6	**RG**	31	124.5 ± 52.9	**QE**	20	118.6 ± 37.2
	**TT**	2	-	**AA**	43	106 ± 36	**AA**	38	106 ± 37	**SS**	6	115.7 ± 35.6	**GG**	2	-	**EE**	0	-
**Glucose**	**CC**	258	5.8 ± 2.3	**GG**	116	5.9 ± 2.6	**GG**	120	5.9 ± 2.6	**AA**	216	5.7 ± 2.1	**RR**	250	5.8 ± 2.3	**QQ**	263	5.7 ± 2.2
**(mmol/L)**	**CT**	23	5.0 ± 0.6	**GA**	123	5.5 ± 1.6	**GA**	125	5.5 ± 1.6	**AS**	61	5.6 ± 1.8	**RG**	31	5.1 ± 0.6	**QE**	20	6.1 ± 2.3
	**TT**	2	-	**AA**	43	5.5 ± 2.3	**AA**	38	5.6 ± 2.5	**SS**	**6**	**8.7 ± 5.4**	**GG**	2	-	**EE**	0	-
**Cholesterol**	**CC**	253	5.3 ± 1.1	**GG**	113	5.3 ± 1.1	**GG**	117	5.3 ± 1.1	**AA**	215	5.3 ± 1.1	**RR**	244	5.3 ± 1.1	**QQ**	258	5.3 ± 1.1
**(mmol/L)**	**CT**	22	5.3 ± 1.3	**GA**	120	5.2 ± 1.1	**GA**	122	5.2 ± 1.1	**AS**	56	5.3 ± 1.1	**RG**	31	4.9 ± 1.1	**QE**	19	5.2 ± 1.5
	**TT**	2	-	**AA**	43	5.4 ± 1.2	**AA**	38	5.4 ± 1.2	**SS**	6	4.9 ± 0.7	**GG**	2	-	**EE**	0	-
**1,25 Vitamin D**	**CC**	249	86.9 ± 41.9	**GG**	115	89.6 ± 43.4	**GG**	118	89.9 ± 43.8	**AA**	209	88.3 ± 42.3	**RR**	241	86.3 ± 44.6	**QQ**	254	86.6 ± 43.5
**(pmol/L)**	**CT**	22	85.2 ± 58.2	**GA**	116	81.5 ± 44.4	**GA**	119	80.5 ± 43.5	**AS**	58	84.2 ± 46.9	**RG**	31	89.9 ± 32.1	**QE**	19	87.4 ± 41.3
	**TT**	2	-	**AA**	41	93.1 ± 38.9	**AA**	36	96.4 ± 38.5	**SS**	6	55.4 ± 31.7	**GG**	1	-	**EE**	0	-
**Systolic blood**	**CC**	259	136 ± 21	**GG**	116	135 ± 20	**GG**	120	135 ± 20	**AA**	217	137 ± 21	**RR**	251	137 ± 20	**QQ**	264	136 ± 21
**pressure**	**CT**	23	133 ± 17	**GA**	124	138 ± 20	**GA**	126	139 ± 21	**AS**	61	134 ± 17	**RG**	31	132 ± 31	**QE**	20	137 ± 17
**(mmHg)**	**TT**	2	-	**AA**	43	131 ± 22	**AA**	38	129 ± 20	**SS**	6	126 ± 10	**GG**	2	-	**EE**	0	-

Results are shown as mean ± SD; −, indicates values not provided. Individual SNP genotypes that were present in N ≤3 individuals were excluded from analysis. Associations at the *P* ≤0.2 statistical level and used for multivariate modelling are highlighted in bold. Calcium, serum total calcium (mmol/L); Phosphate, serum phosphate (mmol/L); PTH, serum intact parathyroid hormone; Creatinine, serum creatinine; Glucose, serum glucose; Cholesterol, serum total cholesterol; 1,25 Vitamin D, 1,25-dihydroxyvitamin D. The genotypic alleles of the A986S, R990G and Q1011E coding region SNPs are represented by amino acids.

The *CASR* SNPs were not significantly associated with AoC or CAC scores at baseline, or with progression of arterial calcification, or the occurrence of cardiovascular events during the study follow-up period (Table 4). Univariate subgroup analyses that excluded patients with diabetes, hyperparathyroidism or hyperphosphataemia, as these factors are key determinants of vascular calcification [30–32], also did not reveal any association between *CASR* SNPs and AoC or CAC scores (S2–S4 Tables). Furthermore, an analysis of *CASR* SNP genotypes in RTRs stratified according to the presence of low or high arterial calcification scores did not reveal any significant associations (Table 5). Using the Kaplan-Meier curve estimate, the effect of *CASR* variants on all-cause mortality was assessed and was shown to be absent for all the *CASR* SNPs (data not shown, *P* >0.05). An analysis of associations between multi-locus *CASR* haplotypes and serum glucose, indices of mineral metabolism and arterial calcification scores, was not performed due to the low prevalence of minor alleles in these haplotypes.

**Table 4:** Univariate analysis of associations between *CASR* SNP genotypes and occurrence of arterial calcification or cardiovascular (CV) events.

	rs115759455			rs7652589			rs1501899			A986S			R990G			Q1011E		
	**Genotype**	**N**	**Value**	**Genotype**	**N**	**Value**	**Genotype**	**N**	**Value**	**Genotype**	**N**	**Value**	**Genotype**	**N**	**Value**	**Genotype**	**N**	**Value**
**Aortic**	**CC**	245	3505 ± 7349	**GG**	110	3274 ± 7904	**GG**	112	3288 ± 7841	**AA**	205	2967 ± 6253	**RR**	235	3447 ± 7245	**QQ**	250	3307 ± 7196
**calcification**	**CT**	20	1052 ± 1855	**GA**	116	3660 ± 6531	**GA**	119	3783 ± 6799	**AS**	56	4800 ± 9724	**RG**	30	2323 ± 5979	**QE**	17	3136 ± 5467
**(AgS)**	**TT**	2	-	**AA**	41	2327 ± 6334	**AA**	36	1715 ± 5282	**SS**	6	508 ± 471	**GG**	2	-	**EE**	0	-
**Coronary artery**	**CC**	244	965 ± 1635	**GG**	110	890 ± 1698	**GG**	112	925 ± 1699	**AA**	205	959 ± 1606	**RR**	234	971 ± 1639	**QQ**	249	902 ± 1568
**calcification**	**CT**	20	703 ± 1162	**GA**	115	1064 ± 1560	**GA**	119	1058 ± 1574	**AS**	56	886 ± 1652	**RG**	30	755 ± 1306	**QE**	17	1477 ± 1989
**(AgS)**	**TT**	2	-	**AA**	41	722 ± 1434	**AA**	36	594 ± 1328	**SS**	6	752 ± 886	**GG**	2	-	**EE**	0	-
**Change in AoC**	**CC**	170	69 ± 2012	**GG**	84	71 ± 1259	**GG**	85	51 ± 1255	**AA**	144	179 ± 1798	**RR**	164	14 ± 1973	**QQ**	177	94 ± 1943
**(AgS)**	**CT**	17	361 ± 1067	**GA**	74	170 ± 2407	**GA**	75	296 ± 2523	**AS**	40	-211 ± 2459	**RG**	21	754 ± 1709	**QE**	10	135 ± 2086
	**TT**	0	-	**AA**	29	-21 ± 2299	**AA**	27	-319 ± 1881	**SS**	3	-	**GG**	2	-	**EE**	0	-
**Change in CAC**	**CC**	170	363 ± 1462	**GG**	84	381 ± 1807	**GG**	85	383 ± 1796	**AA**	144	350 ± 1478	**RR**	164	333 ± 1413	**QQ**	177	357 ± 1431
**(AgS)**	**CT**	17	375 ± 685	**GA**	74	436 ± 1123	**GA**	75	449 ± 1127	**AS**	40	360 ± 1115	**RG**	21	646 ± 1432	**QE**	10	490 ± 956
	**TT**	0	-	**AA**	29	134 ± 371	**AA**	27	74 ± 193	**SS**	3	-	**GG**	2	-	**EE**	0	-
**≥ 1 CV event**	**CC**	259	80 (31%)	**GG**	116	33 (28%)	**GG**	120	34 (28%)	**AA**	217	63 (29%)	**RR**	251	79 (32%)	**QQ**	264	77 (29%)
	**CT**	23	4 (17%)	**GA**	124	41 (33%)	**GA**	126	42 (33%)	**AS**	61	21 (34%)	**RG**	31	5 (16%)	**QE**	20	7 (35%)
	**TT**	2	-	**AA**	43	9 (21%)	**AA**	38	8 (21%)	**SS**	6	0	**GG**	2	-	**EE**	0	-

Aortic calcification (AoC) and coronary artery calcification (CAC) scores are provided at baseline in Agatston units (AgS), and the incremental change in AoC and CAC scores observed at the follow-up visit (after a mean period of 4.4 ± 0.3 years) are also provided. Results are shown as mean ± SD or N (%); −, indicates values not provided. Individual SNP genotypes that were present in N ≤3 individuals were excluded from analysis. The genotypic alleles of the A986S, R990G and Q1011E coding region SNPs are represented by amino acids.

**Table 5:** Comparison of *CASR* SNP genotypes in patients with low and high levels of aortic calcification (AoC) and coronary artery calcification (CAC).

	AoC		CAC	
	<600mg	>600mg	<100mg	>100mg
rs115759455				
CC	185 (91%)	60 (95%)	151 (90%)	94 (95%)
CT	17 (8%)	3 (5%)	15 (9%)	5 (5%)
TT	2 (1%)	0	2 (1%)	0
*P*	-	1.0	-	1.0
rs7652589				
GG	88 (43%)	22 (35%)	74 (44%)	36 (36%)
GA	81 (40%)	35 (56%)	63 (38%)	53 (54%)
AA	35 (17%)	6 (10%)	31 (18%)	10 (10%)
*P*	-	0.40	-	0.15
rs1501899				
GG	88 (43%)	24 (38%)	73 (43%)	39 (39%)
GA	84 (41%)	35 (56%)	66 (39%)	53 (39%)
AA	32 (16%)	4 (6%)	29 (17%)	7 (39%)
*P*	-	0.36	-	0.11
A986S				
AA	158 (77%)	47 (75%)	127 (%)	78 (79%)
AS	40 (20%)	16 (25%)	38 (%)	12 (12%)
SS	6 (3%)	-	3 (%)	3 (3%)
*P*	-	1.0	-	1.0
R990G				
RR	176 (86%)	59 (94%)	147 (88%)	88 (89%)
RG	26 (13%)	4 (6%)	19 (11%)	11 (11%)
GG	2 (1%)	-	2 (1%)	-
*P*	-	1.0	-	1.0
Q1011E				
QQ	192 (94%)	58 (92%)	159 (95%)	91 (92%)
QE	12 (6%)	5 (8%)	9 (5%)	8 (8%)
EE	-	-	-	-
*P*	-	1.0	-	1.0

Results are shown as N (%). P-values (*P*) represent a Chi-squared analysis of the <600mg AoC group (N = 204) versus the >600mg AoC group (N = 63) and <100mg CAC group (N = 168) versus the >100mg CAC group (N = 99), respectively. All values are shown following Bonferroni correction. −, indicates values not provided. The genotypic alleles of the A986S, R990G and Q1011E coding region SNPs are represented by amino acids.

Following Bonferroni correction, all parameters that had associations at the *P ≤*0.2 statistical level were entered into a stepwise multivariate linear regression model, which corrected for potentially confounding influences (Table 6). The homozygous S986 allele continued to remain significantly associated with serum glucose after correcting for the presence of diabetes, as well as correcting for age, gender, body mass index (BMI), renal function, glucocorticoid and immunosuppressant usage, and transplantation vintage (Table 6). A significant association was also observed between the promoter region *CASR* SNP, rs115759455, and serum phosphate concentrations after correcting for confounding parameters such as calcium and vitamin D supplementation, serum creatinine and estimated glomerular filtration rate, serum calcium, 1.25-dihydroxyvitamin D and intact PTH concentrations. Thus, the mean serum phosphate concentration in patients homozygous for the rs115759455 major allele (CC) was 1.00 ± 0.23 mmol/L (N = 258) and 1.14 ± 0.31 mmol/L for patients harbouring the CT alleles (N = 23, *P* <0.05).

**Table 6:** Significant determinants of serum glucose and phosphate concentrations.

Step number	Parameter	B	95% CI	*P*
**Glucose**				
**1**	Diabetes	0.203	0.166 to 0.240	<0.0001
**2**	A986S (SS)	0.126	0.039 to 0.214	<0.05
**Phosphate**				
**1**	eGFR	-0.147	-0.208 to -0.086	<0.0001
**2**	Calcium	-0.851	-1.259 to -0.422	<0.0001
**3**	1,25-dihydroxyvitamin D	-0.077	-0.123 to -0.030	0.001
**4**	rs115759455 (CT)	0.051	0.009 to 0.093	<0.05
**5**	Parathyroid hormone	-0.049	-0.088 to -0.010	<0.05

All parameters scoring *P* ≤0.2 in univariate analyses underwent multivariate modelling and correction for the influence of potentially confounding parameters. Confounding parameters for serum glucose concentration entered into the multivariate stepwise linear regression model were gender, the presence of diabetes, age, body mass index, glucocorticoid and tacrolimus therapy, and transplantation vintage. Serum phosphate concentrations were adjusted for the effect of estimated glomerular filtration rate (eGFR), creatinine, calcium, 1,25-dihydroxyvitamin D, gender, parathyroid hormone concentrations, and calcium and vitamin D supplementation. Multivariate modelling demonstrated the presence of diabetes and the homozygous minor allele of the A986S SNP as independent predictors of serum glucose concentrations. Estimated GFR, serum calcium, parathyroid hormone, 1,25-dihydroxyvitamin D concentrations and the heterozygous form of the rs115759455 SNP were revealed to be independent predictors of serum phosphate concentrations. P-values (*P*) are displayed following Bonferroni correction. B, regression coefficient; CI, confidence interval.

## Discussion

Our study has revealed that the A986S *CASR* SNP is a predictor of serum glucose concentrations independently of BMI and the presence of diabetes mellitus. Thus, patients who were homozygous for the S986 minor allele had elevations in serum glucose, and this highlights a potential role for the CaSR as a regulator of systemic glucose homeostasis. Indeed, this would be consistent with the reported expression of the CaSR in pancreatic islet beta-cells and that CaSR activation by extracellular calcium or calcimimetic drugs in isolated islets can stimulate beta-cell activity and insulin secretion [33–35]. In addition, the CaSR is expressed and functionally active in adipocytes [36], and may potentially regulate the peripheral actions of insulin, as highlighted by the finding of an association with the A986S *CASR* polymorphism and insulin resistance in patients with the polycystic ovarian syndrome [37]. Moreover, altered CaSR function in diabetic patients may contribute to the development of atherosclerosis and increased cardiovascular risk [38].

A significant association was also observed with the rs115759455 5’UTR *CASR* SNP and increased serum phosphate concentrations. Common genetic variants have been previously linked to serum phosphate concentrations, including a SNP (rs17265703) located on chromosome 3q21.1, which was shown to be in strong linkage disequilibrium with the A986S *CASR* variant [39]. Our study revealed the rs115759455 minor *CASR* allele (T) to be an independent predictor of serum phosphate concentrations, as heterozygosity (TC), when compared to homozygosity (CC) for the major allele, was associated with significantly increased serum phosphate concentrations; such effects of homozygosity (TT) for the minor allele could not be established as only two RTRs were homozygous. Circulating phosphate concentrations are regulated by the actions of PTH and fibroblast growth factor-23 (FGF-23) on phosphate reabsorption by the proximal renal tubule, and by 1,25-dihydroxyvitamin D mediated intestinal phosphate reabsorption [40]. Our findings indicate that the CaSR may regulate phosphate homeostasis independently of its effects on circulating PTH and 1,25-dihydroxyvitamin D concentrations. Moreover, studies of mice with the combined ablation of *Casr* and *Pth* alleles indicate that such effects of the CaSR on phosphate homeostasis are also not mediated by FGF-23 [41]. Indeed, the CaSR is likely to have a direct role in regulating circulating phosphate concentrations, as highlighted by micro-perfusion studies of isolated proximal renal tubules, which have revealed CaSR activation in this nephron segment to promote renal phosphate reabsorption [42]. Our findings provide further support for the CaSR being an independent regulator of phosphate metabolism.

However, *CASR* SNPs were not significantly associated with the development and progression of aortic medial calcification or coronary arterial intimal calcification, or cardiovascular outcomes in RTRs. These findings contrast with a report of the A986S *CASR* SNP as an independent predictor of coronary artery disease and cardiovascular mortality [22]. In addition, a mouse model, Nuf, with an activating *Casr* mutation, has been reported to have mineralisation within the aorta, elastic and muscular arteries [43]. The differences in these studies may be partly explained by differences in the cohort characteristics e.g. patient age, ethnicity, medication history, and underlying pathologies of the different patient groups (renal transplant versus non-renal disease); as well as by species differences (man versus mouse). However, these differences may also reflect limitations in our study of the cohort of RTRs, which include the small sample size and low prevalence of some alleles. Moreover, these findings may be affected by survival bias, which favours patients with less severe cardiovascular disease and calcification. Our study of 284 RTRs had a power of 99% and 89% to detect a locus that contributed 10% or 5%, respectively, of the genetic variance, assuming a type 1 error of 0.01 and marker frequency of 0.2. This indicates that our study was sufficiently powered to detect effects that would explain up to 5% of the variance, but not 1% of variance [28]. Nevertheless, the findings of this study indicate that the six common *CASR* polymorphisms are unlikely to play a major role in the development or progression of aortic or coronary artery calcification in patients with renal transplants.

In conclusion, our investigation of associations between common *CASR* variants, arterial calcification and other cardiovascular risk factors in a cohort of renal transplant recipients indicates that these *CASR* variants are unlikely to represent major determinants for calcification of either the aorta or coronary arteries. However, the CaSR was demonstrated to be an independent predictor of serum glucose and phosphate concentrations, thereby highlighting potential new metabolic roles for this GPCR.

## Supporting Information

S1 Dataset Clinical, biochemical, radiological and genotype data for Brussels Renal Transplant Cohort. (XLS)

S1 Table Influence of cumulative steroid dosage on serum glucose concentrations in the Brussels Renal Transplant Cohort. (DOCX)

S2 Table Univariate analysis of associations between *CASR* SNP genotypes and aortic and coronary artery calcification in patients without hyperparathyroidism. (DOCX)

S3 Table Univariate analysis of associations between *CASR* SNP genotypes and aortic and coronary artery calcification in patients without hyperphosphataemia. (DOCX)

S4 Table Univariate analysis of associations between *CASR* SNP genotypes and aortic and coronary artery calcification in non-diabetic patients. (DOCX)
