# Supplementary material for: Association Studies of Calcium-Sensing Receptor (CaSR) Polymorphisms with Serum Concentrations of Glucose and Phosphate, and Vascular Calcification in Renal Transplant Recipients
Source: PLoS One. 2015 Mar 18;10(3):e0119459. doi: 10.1371/journal.pone.0119459 (PMC4364904; doi:10.1371/journal.pone.0119459)
Supplement: S1 Table — (DOCX) [file pone.0119459.s002.docx]

**S1 Table.** Influence of cumulative steroid dosage on serum glucose concentrations in the Brussels Renal Transplant Cohort.

| Cumulative prednisolone dose  (mg) | Number of patients  (%) | Serum glucose concentration (mmol/L) |
| --- | --- | --- |
| < 5000 | 69 (24%) | 5.85 ± 2.51 |
| 5000-15000 | 84 (30%) | 5,95 ± 2.43 |
| 15000-25000 | 56 (20%) | 5.57 ± 1.99 |
| > 25000 | 75 (26%) | 5.43 ± 1.67 |

Serum glucose concentrations are shown as mean ± SD. Analysis by one-way ANOVA did not detect any significant associations between cumulative steroid dosage and serum glucose concentrations.
