# Supplementary material for: Association Studies of Calcium-Sensing Receptor (CaSR) Polymorphisms with Serum Concentrations of Glucose and Phosphate, and Vascular Calcification in Renal Transplant Recipients
Source: PLoS One. 2015 Mar 18;10(3):e0119459. doi: 10.1371/journal.pone.0119459 (PMC4364904; doi:10.1371/journal.pone.0119459)
Supplement: S4 Table — (DOCX) [file pone.0119459.s005.docx]

**S4 Table.** Univariate analysis of associations between *CASR* SNP genotypes and aortic and coronary artery calcification in non-diabetic patients.

|  | **rs115759455** | | | **rs7652589** | | | **rs1501899** | | | **A986S** | | | **R990G** | | | **Q1011E** | | |
| --- | --- | --- | --- | --- | --- | --- | --- | --- | --- | --- | --- | --- | --- | --- | --- | --- | --- | --- |
|  | **Genotype** | **N** | **Value** | **Genotype** | **N** | **Value** | **Genotype** | **N** | **Value** | **Genotype** | **N** | **Value** | **Genotype** | **N** | **Value** | **Genotype** | **N** | **Value** |
| **Aortic** | **CC** | 209 | 3158 ± 7200 | **GG** | 93 | 3429 ± 8500 | **GG** | 94 | 3425 ± 8460 | **AA** | 177 | 2568 ± 5836 | **RR** | 199 | 3139 ± 7113 | **QQ** | 216 | 2982 ± 7027 |
| **calcification** | **CT** | 19 | 1006 ± 1894 | **GA** | 100 | 3113 ± 5904 | **GA** | 103 | 3060 ± 5828 | **AS** | 48 | 4652 ± 10054 | **RG** | 28 | 1934 ± 5673 | **QE** | 13 | 2703 ± 5147 |
| **(AgS)** | **TT** | 1 | - | **AA** | 36 | 1362 ± 4552 | **AA** | 32 | 1315 ± 4775 | **SS** | 4 | 351 ± 478 | **GG** | 2 | - | **EE** | 0 | - |
| **Coronary artery** | **CC** | 208 | 913 ± 1670 | **GG** | 93 | 850 ± 1751 | **GG** | 94 | 865 ± 1739 | **AA** | 176 | 885 ± 1612 | **RR** | 198 | 929 ± 1682 | **QQ** | 215 | 866 ± 1622 |
| **calcification** | **CT** | 19 | 661 ± 1179 | **GA** | 99 | 991 ± 1572 | **GA** | 102 | 996 ± 1600 | **AS** | 48 | 91 ± 1766 | **RG** | 28 | 663 ± 1271 | **QE** | 13 | 1249 ± 1814 |
| **(AgS)** | **TT** | 1 | - | **AA** | 36 | 701 ± 1488 | **AA** | 32 | 612 ± 1395 | **SS** | 4 | 754 ± 1005 | **GG** | 2 | - | **EE** | 0 | - |
| **Change in AoC** | **CC** | 148 | -22 ± 2018 | **GG** | 72 | 72 ± 1304 | **GG** | 72 | 57 ± 1306 | **AA** | 125 | 119 ± 1765 | **RR** | 142 | -57 ± 2004 | **QQ** | 156 | 40 ± 1964 |
| **(AgS)** | **CT** | 16 | 402 ± 1088 | **GA** | 66 | 101 ± 2488 | **GA** | 67 | 126 ± 2469 | **AS** | 36 | -338 ± 2543 | **RG** | 20 | 579 ± 1544 | **QE** | 8 | -369 ± 1683 |
|  | **TT** | 0 | - | **AA** | 26 | -328 ± 1920 | **AA** | 25 | -371 ± 1946 | **SS** | 3 | 177 ± 386 | **GG** | 2 | - | **EE** | 0 | - |
| **Change in CAC** | **CC** | 148 | 353 ± 1547 | **GG** | 72 | 380 ± 1938 | **GG** | 72 | 389 ± 1937 | **AA** | 125 | 333 ± 1562 | **RR** | 142 | 332 ± 568 | **QQ** | 156 | 357 ± 1510 |
| **(AgS)** | **CT** | 16 | 401 ± 699 | **GA** | 66 | 446 ± 1168 | **GA** | 67 | 431 ± 1161 | **AS** | 36 | 382 ± 1173 | **RG** | 20 | 579 ± 1435 | **QE** | 8 | 364 ± 896 |
|  | **TT** | 0 | - | **AA** | 26 | 71 ± 194 | **AA** | 25 | 71 ± 198 | **SS** | 3 | 1117 ± 1781 | **GG** | 2 | - | **EE** | 0 | - |

Patients that had a history of diabetes (N = 43 patients) were excluded from an analysis of *CASR* SNP genotypes and aortic calcification (AoC) and coronary artery calcification (CAC) scores. AoC and CAC scores are provided at baseline in Agatston units (AgS), and the incremental change in calcification scores observed at the follow-up visit (after a mean period of 4.4 ± 0.3 years) are also provided. Results are shown as mean ± SD; -, indicates values not provided. Individual SNP genotypes that were present in N ≤ 3 individuals were excluded from analysis. The genotypic alleles of the A986S, R990G and Q1011E coding region SNPs are represented by amino acids.
